# First Baseline of Circulating Genotypic Lineages of *Mycobacterium tuberculosis* in Patients from the Brazilian Borders with Argentina and Paraguay

**DOI:** 10.1371/journal.pone.0107106

**Published:** 2014-09-09

**Authors:** Luzia Neri C. Machado, Nadir R. Marcondes, Clarice Q. Fijimura Leite, Adolfo C. Barreto Santos, Fernando Rogério Pavan, Vanessa Pietrowski Baldin, Aline Lemes Castilho, Vera Lúcia D. Siqueira, Lilian Cristiane Baeza, Henri Berghs, Rosilene Fressatti Cardoso

**Affiliations:** 1 Postgraduate Program in Biosciences and Physiopatology, State University of Maringá, Maringá, Paraná, Brazil; 2 Center of Medical and Pharmaceutical Sciences, State University of Western Paraná, Cascavel, Paraná, Brazil; 3 School of Pharmaceutical Sciences, Department of Biological Sciences, Paulista State University, Araraquara, São Paulo, Brazil; 4 Department of Clinical Analysis and Biomedicine, State University of Maringá, Maringá, Paraná, Brazil; 5 Fairport Ltda, São Paulo, Brazil; St. Petersburg Pasteur Institute, Russian Federation

## Abstract

**Background:**

At the triple border Brazil/Paraguay/Argentina there is easy mobility from one city to another for economic and tourism activities. This constant and fast population mobility is mainly to visit Iguazu Falls, in the Iguazu River, on the border of the Brazilian state of Paraná and the Argentina. As the incidence of tuberculosis is high in this setting, our study aimed to establish a first baseline of circulating genotypic lineages of *Mycobacterium tuberculosis*.

**Methodology/Principal Findings:**

This study included 120 patients from 10 cities in southwestern Paraná, Brazil with pulmonary symptoms, from July 2009 to July 2011. Information about sex, age, clinical features and address was collected by reviewing the national tuberculosis notification database. Of these, 96 (80%) isolates were identified as *M. tuberculosis* and 22 (22.9%) were drug resistant (20, 20.8% INH mono-resistant and 2, 2.1% multidrug-resistant). All isolates were subjected to genotyping by Spoligotyping and MIRU-VNTR typing. The distribution of the isolates analyzed by spoligotyping revealed 30 distinct patterns. The four mainly detected clades were Latin American and Mediterranean (LAM), ill-defined T, Haarlem (H) and S. The MIRU-VNTR showed 85 distinct patterns. Spoligotyping combined to MIRU-VNTR allowed 90 distinct patterns.

**Conclusions/Significance:**

Our study demonstrated that there is significant molecular diversity in circulating *M. tuberculosis*, with predominance of the LAM and T clades in cities of southwestern Paraná, Brazil, bordering Argentina and Paraguay.

## Introduction

Tuberculosis (TB) is an infectious disease that remains a major public health problem in the world. Brazil is among 22 countries with major number of cases of TB in the world, with an incidence of 46/100,000 populations in 2012 and the distribution of TB cases varies greatly according to distinct geographic regions [1]. In the state of Paraná (PR), south of Brazil, 2,350 new TB cases (incidence 22.4/100,000) were detected in 2011 [2]. However, these rates are higher in certain regions in the state of Paraná, such as in Paranaguá City, a port area, and on the triple border of Brazil/Paraguay/Argentina. The three municipalities concentrated near the border, which are Foz do Iguaçu in Brazil, Ciudad del Este in Paraguay and Puerto Iguazu in Argentina, have an incidence of 56.6/100,000, 42.5/100,000, and 23.4/100,000, respectively [3]. These high rates are related to the living conditions of populations, lack of access to health services in some regions and high population mobility. Economic activities and tourism to see one of the New Seven Wonders of Nature, Iguazu Falls (Cataratas do Iguaçu), a waterfall on the Iguazu River on the border of the Brazilian state of Paraná and Misiones in Argentina, have provided strong population growth in Brazilian cities near to the border. In this setting, the constant and fast population mobility from one city to another can favors the spread of TB. In the two major municipalities at the Brazilian border, Foz do Igauçu and Cascavel, which have laboratory to perform culture for *Mycobacterium tuberculosis* and attend many others small surrounding cities for TB diagnosis had an incidence of TB 41.8/100,000 and 25.5/100,000 respectively.

Molecular typing methods have been used for a variety of epidemiological investigations and fast tracking geographic distribution of *M. tuberculosis* clusters [4]. Identical strains fingerprints are called clusters and are usually associated with recent transmission, while strains presenting unique fingerprint profile suggest remote transmission [5], [6]. A PCR-based method, Mycobacterial Interspersed Repetitive Unit - Variable Number Tandem Repeat (MIRU-VNTR) typing allows the high throughput and discriminatory analysis of *M. tuberculosis* clinical isolates [7], [8]. The 15- and 24-MIRU-VNTR have been indicated for epidemiological and phylogenetical studies with *M. tuberculosis*. However, the 12-MIRU-VNTR added of Spoligotyping, another PCR based method, has been widely used in the molecular epidemiology of TB. Spoligotyping investigates the population structure of *M. tuberculosis*, focusing on the identification of genotypic lineages, spoligotype clades and their geographic distribution [9]. Spoligotyping has proven its usefulness for studying phylogeographical aspects of prevailing *M. tuberculosis* genotypic lineages and in combination with 12-MIRU-VNTR, it is highly discriminatory allowing the study of both the epidemiological and phylogeographical aspect of tubercle bacilli, including large-scale studies [10], [11].

This study aimed to establish a first molecular insight of circulating genotypic lineages of *M. tuberculosis* in cities in the southwestern Paraná State, south of Brazil, which borders the countries of Paraguay and Argentina countries, based on Spoligotyping and 12-MIRU-VNTR typing.

## Results

### Patients, bacterial isolates and drug susceptibility test

Of 96 *M. tuberculosis* identified, 64 (66.7%) were isolated from male patients, which account for a male/female ratio of about 2∶1 (M/F = 64/32). The age of patients ranged from 18 to 82 years old (36.75±13.38) and an age distribution of: 17–19 years, 4 (4.1%); 20–29 years, 25 (26.0%); 30–39 years, 35 (36.7%); 40–49 years, 18 (18.7%); 50–59 years, 5 (5,2%) and ≥60 years, 9 (9.3%). Drug susceptibility testing was performed on all 96 isolates and resistance was detected in 22 (22.92%); 20 (20.8%) were INH mono-resistant and 2 (2.1%) multidrug-resistant (MDR-TB) (Table 1). All resistant *M. tuberculosis* isolates were from patients residing in the municipality of Cascavel, Parana, Brazil. Four of these isolates were obtained from prisoners.

**Table 1:** Sex and age of patients and drug susceptibility, Spoligotyping and MIRU patterns of 96 *Mycobacterium tuberculosis* isolates from cities in southwestern Paraná, Brazil.

isolates	Age	Sex	DST	Spoligotyping			MIRU	
				Spoligotypes	Family	SIT	MIRU types	MIT
1	30	M	H	777777607760731	LAM4	60	124326153324	246
2	21	M	S	757757607760771	LAM9*	/	124326143321	/
3	30	M	H	777777607760731	LAM4	60	124326163324	/
5	26	M	H	757777607760771	LAM9	822	124326143222	/
6	26	M	H	777777607760771	LAM9	42	124326153326	816
7	36	M	H	757777607760771	LAM9	822	124326153222	201
8	38	M	H	757777607760771	LAM9	822	124326153322	/
9	45	M	S	777777777760771	T1	53	324226153321	/
11	25	M	H	777777777760731	T2	52	024326143324	/
12	27	M	H	777777607760731	LAM4	60	324326143324	/
13	30	M	H	777777607760731	LAM4	60	024326143324	/
14	41	M	S	777777777760771	T1	53	324125153321	/
15	31	M	S	777777777760731	T2	52	324326143324	/
16	30	M	H	777777777760731	T2	52	324326143324	/
17	25	M	S	777777777760771	T1	53	124326153324	246
18	27	F	S	777777777760771	T1	53	224325153323	33
19	61	F	S	777777777760771	T1	53	124326143326	/
20	31	M	S	777777607760771	LAM9	42	224221143223	/
21	60	M	H	777777607760771	LAM9	42	124326153224	140
22	37	M	S	777777607760771	LAM9	42	024326143324	/
23	49	F	S	377777607760771	LAM9	177	123226143321	/
24	64	M	S	777777607760771	LAM9	42	124316153226	/
25	39	M	S	377777607760771	LAM9	177	224226143321	738
26	23	F	S	377777607760771	LAM9	177	224226153321	25
27	24	M	H	777777607760771	LAM9	42	223326153323	236
28	28	F	S	777777607760771	LAM9	42	124326143225	/
29	32	M	S	777777607760771	LAM9	42	123226153321	/
30	18	F	S	377777607760771	LAM9	177	123226153321	/
31	50	M	S	377777677760771	T1*	/	123216153321	/
33	24	F	S	377777677760771	T1*	/	123226153321	/
34	43	M	H	777777777760771	T1	53	122222153321	/
36	38	M	S	577777777760771	T1	334	223326153326	19
37	18	M	S	763777777760771	T1	713	125326154323	/
38	28	F	S	777777777760771	T1	53	123223173533	/
39	72	F	MDR	777777607760731	LAM4	60	144326143324	/
41	32	F	S	000377637760420	S*	/	224316173228	/
42	23	M	S	777777607760731	LAM4	60	224326143324	39
43	22	M	H	777777607760731	LAM4	60	124326143324	519
44	64	F	S	777777607760771	LAM9	42	124226153322	/
45	48	F	S	757777607560771	LAM9*	/	124326132223	/
46	37	M	S	777777774020771	H1	47	224326132323	/
47	41	M	S	777777777760771	T1	53	123323132321	/
48	35	M	H	777777607760771	LAM4	60	223326132424	/
49	34	M	S	777777607760731	LAM4	60	223326132324	/
50	28	F	S	777777607560771	LAM6*	/	124326132222	/
51	42	F	MDR	777777607760731	LAM4	60	124326132324	/
52	42	M	H	777777777760771	T1	53	224313132324	/
53	41	F	H	777777607760731	LAM4	60	124326132324	/
54	25	M	S	777777777760771	T1	53	224326132324	/
55	30	F	S	777777607760731	LAM4	60	123326132324	/
56	39	M	S	777777607560771	LAM6	64	121426132224	/
57	61	F	S	577777607760771	LAM9	866	123326132321	/
58	37	M	S	377777607760771	LAM9	177	224226132321	/
59	28	M	S	777777607760771	LAM9	42	224236552326	/
60	37	M	S	377777607760771	LAM9	177	224236152321	/
61	21	M	S	000057607760000	X3*	/	223236162228	/
62	54	M	S	040054604020771	LAM8*	/	225333550323	/
63	22	M	S	377777607760771	LAM9	177	221236553321	/
64	62	M	S	377777607760771	LAM9	177	224236553321	/
65	30	F	S	777777607760731	LAM4	60	125336543324	/
66	58	M	S	377777607760771	LAM9	177	224236553321	/
67	58	F	S	777777607760731	LAM4	60	123336143324	/
68	37	M	S	777774047560751	H37Rv*	/	123336523322	/
69	30	F	S	777764006020651	LAM8*	/	223-36123522	/
70	26	F	S	040000004020771	H2*	/	123333523323	/
71	27	F	S	040000004020771	H2*	/	124313553323	/
72	40	M	H	777777764760731	T1*	/	223326553224	/
73	66	M	S	777777647760771	T1	2298	124326543325	/
76	54	M	S	776377777760771	S	34	234325153324	/
77	35	F	H	777777607760731	LAM4	60	124326143324	519
78	43	M	S	777777760720771	H*	/	224325153321	161
79	32	M	S	777777607760771	LAM9	42	225406143221	/
82	30	M	S	777777607560771	LAM6*	/	123206153122	/
83	30	M	S	777777607560771	LAM6*	/	124306153122	/
85	29	F	S	377777607760771	LAM9	177	224306153128	/
86	34	F	S	377777607760771	LAM9	177	224306153128	/
87	30	M	H	777777607760731	LAM4	60	124306153324	/
93	38	F	S	777777607760771	LAM9	42	224206153321	/
94	49	F	S	577777607760771	LAM9	866	124306153326	/
95	82	M	S	777777607760771	LAM9	42	223205173324	/
96	38	M	S	777777777760771	T1	53	223405143322	/
97	20	M	S	777777777760731	T2	52	124306143324	/
98	22	M	S	677737677760661	T1*	/	213205173321	/
99	45	M	S	777777777760771	T1	53	224303143323	/
100	25	M	S	776377777760771	S	34	233305143324	/
104	40	M	S	677777777760771	T1	196	214205163321	/
107	43	M	S	776377777760771	S	34	233325153224	218
109	17	F	S	677777607760771	LAM1	20	224226143321	738
110	61	M	S	776377777760771	S	34	233325153334	/
111	25	F	S	777777600000331	LAM8*	/	123326152224	/
112	42	M	S	777777607720621	LAM9*	/	224317153227	/
115	31	F	S	777777774020771	H1	47	225313153323	42
117	31	F	S	777777607760771	LAM9	42	224216143221	/
118	31	M	S	777777607760731	LAM4	60	124026143321	/
119	45	M	S	777777607760771	LAM9	42	123326143226	/
120	22	F	S	776377777760771	S	34	233325153224	218

M: male; F: female; Drug susceptibility testing, DST; H, isoniazid resistant; MDR, isoniazid and rifampicin resistant; S, susceptible to isoniazid, rifampicin and pyrazinamide; SIT, Shared International Types; MIT, MIRU international types; -, no amplification; /, not described in SITVITWEB database; * family assignment by SPOTCLUST.

### Spoligotyping and MIRU-VNTR typing

The distribution of 96 *M. tuberculosis* isolates analyzed by spoligotyping revealed 30 distinct patterns (n = 77, 80.2% isolates) and 15 (n = 19, 19.8% isolates) that have not been described in SITVITWEB database yet. According to SITVITWEB database, 71/77 (92.2%) isolates were clustered into nine Shared International Types (SIT), comprising from 2 to 17 isolates each cluster and the remaining 6 (7.8%) isolates showed unique SIT. The main SITs found in the present study were SIT60 (n = 17, 17.7%), SIT42 (n = 15, 15.6%), SIT53 (n = 12, 12.5%), and SIT177 (n = 11, 11.5%). The 77 (80.2%) isolates, identified in the SITVITWEB database, had been classified into clades and sublineages, which ranked in four clades, Latin American and Mediterranean (LAM) clade and sublineages, ill-defined T clade and sublineages, S and Haarlem (H) clades with 50 (52.1%), 20 (20.8%), 5 (5.2%) and 2 (2.1%) isolates, respectively (File S1).

Out of 19 (19.8%) isolates none identified in SITVITWEB database, 11 (11.5%) had unique SIT and the remaining 8 (8.3%) were included in 4 clusters, comprising 2 isolates for each one (Table 1). The clades assignment for these isolates, by SPOTCLUST, revealed that 9 (47.4%) belonged to the LAM, 4 (21.1%) to T and 3 (15.8%) to H. There were three other isolates pertaining to X, S and H_37_Rv (Table 1).

12 loci-MIRU-VNTR typing conducted in the 96 clinical isolates showed a total of 85 distinct MIRU patterns (File S1). Seventy-six (79.2%) isolates had unique patterns and the remaining 20 (20.8%) were included in 9 clusters comprising 2 to 3 isolates for each one (Table 1). Out of 96 isolates, 18 were identified in the SITIVITWEB database pertaining to MIRU international types (MITs) 19, 25, 33, 39, 42, 140, 161, 201, 218, 236, 246, 519, 738 and 816 (Table 1).

Allele polymorphism analysis of 12 loci-MIRU-VNTR by Hunter*-*Gaston Discrimination Index (HGDI) [22], revealed that MIRU locus 40 was the most discriminatory with 8 alleles, followed by MIRU locus 26 with 6 alleles. In the MIRU locus 40 the presence of single copy and 4 alleles were the most frequent. MIRU loci 2, 10, 16, 20, 23, 27 and 31 were moderately discriminant. MIRU loci 4, 24 and 39 were the poorly discriminant (Table 2). The amplification of MIRU *locus* 16 of isolate 69 was not possible.

**Table 2:** Allele polymorphism by 12 loci-MIRU-VNTR of 96 *Mycobacterium tuberculosis* from cities in southwestern Paraná, Brazil.

MIRU no.	Allele no.									HGI a	Conclusion
	0	1	2	3	4	5	6	7	8		
MIRU 2	3	46	42	5						0.581	Moderately discriminant
MIRU 4		2	89	5						0.139	Poorly discriminant
MIRU 10		2	1	29	59	5				0.533	Moderately discriminant
MIRU 16	1	1	26	65	3					0.499	Moderately discriminant
MIRU 20	15	8	61	12						0.555	Moderately discriminant
MIRU 23		1	1	8		12	73	1		0.404	Moderately discriminant
MIRU 24		85				11				0.205	Poorly discriminant
MIRU 26			3	14	29	43	3	4		0.690	Highly discriminant
MIRU 27		1	18	76	1					0.342	Moderately discriminant
MIRU 31		4	19	70	1	2				0.431	Moderately discriminant
MIRU 39			94	2						0.021	Poorly discriminant
MIRU 40		26	10	12	34	2	7	1	4	0.775	Highly discriminant

a HGI: Hunter-Gaston index.

The allelic diversity of the loci was classified as highly discriminant (HGI >0.6), moderately discriminant (0.3≥ HGI ≤0.6) and poorly discriminant (HGI <0.3).

All 96 *M. tuberculosis* isolates analyzed by spoligotyping and 12-MIRU-VNTR typing combined could be genotypically differentiated in 90 patterns. In this analysis, 84 (87.50%) isolates showed distinct genotypes and 12 (12.50%) were included into six clusters (2 isolates each with 100% similarity) (Table 3).

**Table 3:** Clusters by Spoligotyping/MIRU combination and epidemiological data of 12 *Mycobacterium tuberculosis* isolates from patients in cities in southwestern Paraná, Brazil.

Isolates	spoligotyping	MIRU-VNTR	Date of culture/microscopy of Baciloscopy	DST	Epidemiologic relationships
15	777777777760731	324326143324	11/25/2009	S	No epimiological relation
16	777777777760731	324326143324	07/30/2010	H	No epimiological relation
43	777777607760731	124326143324	06/28/2010	H	Live in the same neighborhood and nearby street
77	777777607760731	124326143324	11/28/2010	H	
51	777777607760731	124326132324	07/25/2010	MDR	No epimiological relation
53	777777607760731	124326132324	07/30/2010	S	No epimiological relation
64	377777607760771	224236553321	09/20/2010	S	Live in neighboring cities
66	377777607760771	224236553321	10/20/2010	S	
85	377777607760771	224306153128	29/12/2010	S	No epimiological relation
86	377777607760771	224306153128	13/01/2011	S	No epimiological relation
107	776377777760771	214205163321	17/05/2010	S	No epimiological relation
120	776377777760771	233325153224	10/05/2011	S	No epimiological relation

DST, Drug susceptibility testing; H, isoniazid resistant; MDR, isoniazid and rifampicin resistant; S, susceptible to isoniazid, rifampicin and pyrazinamide.

## Discussion

This is the first study conducted to establish the circulating genotypic lineages of *M. tuberculosis* isolated from TB patients in southwestern Paraná, Brazil that borders the countries of Paraguay and Argentina. Economic activities and tourism in the triple border have provided strong population growth in some cities from the Brazil border, mainly in Cascavel and Foz do Iguaçu. Constant population mobility and the rapid migration for commercial purpose, from one city to another one, at the triple border, may favor the spread of TB in this setting.

The age and sex distribution of patients in our study reflects the predominance of TB among adult male people (81.4% of all patients) as observed in all other regions in the word [1]. The 2∶1 male to female sex-ratio may be explained by the local socio-cultural barriers. In this setting, women stay mainly at home and men are engaged in external activity, which leads the latter to a higher exposition to the bacillus. In the present study, the prevalent isolates came from Cascavel, Foz de Iguaçu and other surrounding small cities.

The LAM, T and H were the largest clades observed in the present study, the three genotypic clades most frequent in Africa, Central America, Europe and South America [10]. The present results are similar to a previous one, carried out in our laboratory, by Noguti et al. [12], which had greater variability in SITs, but LAM, H and T were the largest clades in the northwestern Paraná. In Araraquara, São Paulo State, which borders northern Paraná, Mendes et al. [13] identified 73% of spoligopatterns in SpolDB4, and LAM, T and H clades were the most prevalent too. More recently, Santos et al. [11], in a comparative study between 1998–2001 and 2002–2006, observed replacement of ill-defined T family for LAM in Araraquara, São Paulo. Furthermore, there are a number of lineages found in Northern Paraná and São Paulo States studies, including Beijing, which was not detected in the present study.

The most prevalent SITs in southwestern Paraná were 60 (prototype of LAM4), 42 (prototype of LAM9), 53 (prototype of T1) and 177 (prototype of LAM9). These four most prevalent SITs observed in our study were found in Argentina too, as reported in SITVITWEB and SpolDB4, but no reference to Paraguay was made [10].

The SITs 334 and 713 (both susceptible and prototype of T1), characterized by one isolate for each one, have not been described in Paraguay and Argentina yet. The first one has been described in Brazil only once, but the second hasn't been yet (http://www.pasteur-guadeloup.fr:8081/SITVITDemo/). The other spoligopatterns, which were described in SITVITWEB database, were already identified in Brazil and some of them in Argentina and Paraguay. The SITs 52 (prototype of T2), 60 (prototype of LAM4), 196 (prototype of T1), 822 (prototype of LAM9) and 866 (prototype of LAM9) were described for the first time in Paraná, Brazil (http://www.pasteur-guadeloup.fr:8081/SITVITDemo/). Of interest, INH mono-resistant was predominant in isolates genotyped as SIT60 (LAM4, 9 isolates), which is frequent in the Americas and some countries in Europe (http://www.pasteur-guadeloup.fr:8081/SITVITDemo/). The two MDR were genotyped as SIT 60 (LAM4) but had different MIRU patterns.

**Figure 1:**
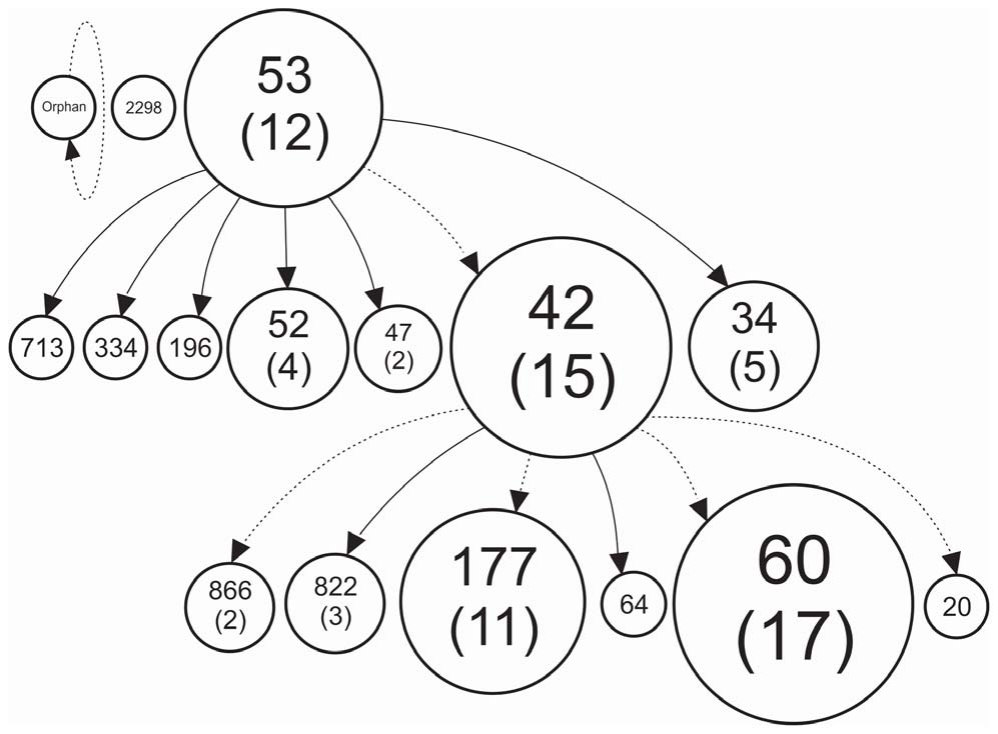
Spoligoforest tree of 96 *M. tuberculosis* clinical isolates from cities in the southwestern Paraná, Brazil, showing all spoligotyping clades represent changes (loss of spacers) by lane. Edges between nodes reflect evolutionary relationships between spoligotypes with arrowheads pointing to descendants. The area of each node is proportional to the number of isolates.

The spoligoforest tree indicates that there are conserved genotypes as the SIT53 (prototype of T1), which demonstrated the ability to generate 7 other genotypes, by the loss of 1 to 6 spacers, which predominate T clade. We could observe with SIT42 (prototype of LAM9) that the loss of only one spacer, in a different section of DR region, yielded 6 different SITs (20, 60, 64, 177, 822, 866) belonging to the LAM clade (figure 1).

We observed a total of 9 single locus variation (SLV) events within the 12-locus subset. The best discriminatory power was obtained considering that a unique SLV in any one of the loci analyzed, remains strongly predictive of the absence of a link between the isolates. According to Hunter and Gaston [22] the most allelic diversity observed in the *M. tuberculosis* isolates studied was in MIRU loci 40 and 26 and moderated polymorphism were found in MIRU loci 2, 10, 16, 20, 23, 27 and 31. These allelic diversities also differs from the obtained in northwestern Paraná by Noguti et al. [12] that observed most allelic diversity in MIRU loci 40, 23, 10 and 16 and moderate polymorphism in MIRU loci 26, 20 and 31. Sharma et al. [14] working with only 6 loci-MIRU (MIRU 4, 10, 16, 26, 39 and 40) observed the most discriminatory loci, in order of diversity, 26, 10, 16 and 40 in isolates from Kanpur, India. Kovalev et al. [15] observed most allelic diversity in MIRU loci 26, 31 and 10 in *M. tuberculosis* isolated in Ural region, Russian Federation. On the other hand these authors refer to MIRU locus 23 as having the lowest discriminatory power, differing from our results, which was MIRU 39.

Despite of high discriminatory power of MIRU typing observed in our study, there were isolates clustered by this method. In these cases, some clustered isolates (25, 109, 29, 30, 1, 17, 11, 13 and 22) were discriminated by spoligotyping, demonstrating the need of using these two techniques combined to provide a higher discriminatory power. In this sense, Spoligotyping combined to 12 loci-MIRU-VNTR allowed 90 different molecular patterns for all 96 *M. tuberculosis* isolates analyzed. In these patterns there were 12 isolates that weren't possible to be differentiated as unique and were included in six patterns with two isolates each one. Four these clustered isolates are of particular concern to be discussed. The isolates 43 and 77, which were INH mono-resistant, were obtained from patients, who lived in the same neighborhood and nearby streets and were referred to the central laboratory in Cascavel for TB laboratorial diagnosis in the same year. Two other isolates (64 and 66) with the same spoligopattern and MIRU, were obtained from patients that lived in neighboring cities (Cascavel and Campo Bonito) and were referred to the central laboratory in Cascavel for TB laboratorial diagnosis. Thus, to overcome the observed limitation, additional study should be considered by using optimized 15- and 24- loci-MIRU typing to differentiate the isolates and provide additional data about any isolates relationship.

In conclusion, our study offers the first baseline about the genetic diversity of *M. tuberculosis* isolated from patients with TB in cities in southwestern Paraná State, Brazil, which borders two other countries, Paraguay and Argentina. This kind of study is important for tracing relationships among the strains and recognition of the mainly clades responsible for spreading the disease. One limitation of the present study is the small number of isolates from some cities in the samples. However this issue will be addressed by additional and better designed studies in this setting, with a higher timescale and number of isolates conducted in the future to understand the chain of TB transmission in this region.

## Materials and Methods

### Patients, bacterial isolates and drug susceptibility test

**Figure 2:**
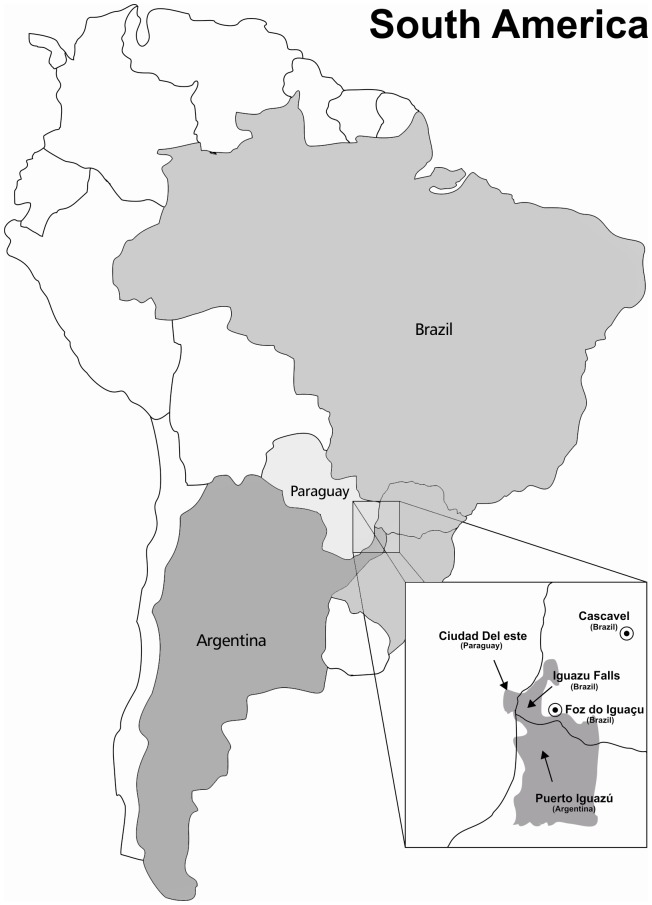
The triple border Brazil/Paraguay/Argentina, with the location of the cities of Foz do Iguacu and Cascavel (Brazil), Ciudad del Este (Paraguay) and Puerto Iguazú (Argentina).

The study was approved by the Ethics Committee of the State University of Maringa, Parana (protocol No. 018/2011, CAAE No. 0375.0.093.000-10). The information about sex, age, clinical features and address of patients was collected by reviewing the national TB notification database. As the study was conducted with *M. tuberculosis* isolated from sputa that were disposed of, in accordance with internal biosecurity rules, after test results release to the patient, and no additional patients' sputa samples and information, which could to identify them were requested (identification was carried out by a number given to each patient), the document participant consents achievement were exempted by the ethics committee. The study included 120 patients (66.7% male patients, age range from 17 to 82 years old) from 10 cities in southwestern Paraná, Brazil, which borders Paraguay and Argentina countries (Figure 2), with confirmed pulmonary TB by microscopy (Ziehl-Neelsen - ZN) and culture (Ogawa medium – OK). All patients were attended at two central laboratories in Cascavel and Foz do Iguaçu cities, southwestern Paraná, from July 2009 to July 2011. Of these, 96 isolates (80%) were identified as *M. tuberculosis* by biochemical methods [16] and drug susceptibility tests (DSTs) were carried out for Isoniazid (INH), Rifampicin (RMP) and Pyrazinamide (PZA) by Löwenstein-Jensen (L-J) Proportion Method [17].

### Spoligotyping and MIRU-VNTR typing

*M. tuberculosis* DNA of 96 *M. tuberculosis* isolates were extracted from cultured cells as described by Cardoso et al. [18] and subjected to genotyping by spoligotyping to detect presence or absence of 43 spacers by using the standard method [9], [19] and by the original 12-MIRU-VNTR loci (2, 4, 10, 16, 20, 23, 24, 26, 27, 31, 39 and 40) as described by Supply et al. [7], [20]. MIRU allele scoring was determined according to Mazars et al. [21] and Supply et al. [8]. The results from each of the 12 loci were combined to create 12-digit allelic profiles. The power of discriminating 12 loci was determined by Hunter*-*Gaston Discrimination Index (HGDI) [22].

### Database analysis

The spoligo and 12-loci-MIRU patterns were compared to international database SITIVITWEB (Institute Pasteur de la Guadalupe) [23], which is an updated version of the published SpolDB4.0 database [10] and available at www.pasteur-guadeloupe.fr:8081/SITVITDemo/. The spoligopatterns not identified in SITVITWEB database were assigned in families by SPOTCLUST at http://cgi2.cs.rpi.edu/~bennek/SPOTCLUST.html. For additional analysis a phylogenetic tree (spoligoforest) was constructed with data from spoligotyping using SpolTools at http://www.emi.unsw.edu.au/spolTools/.

BioNumerics software (version 4.45; Applied Maths, Sint-Martens-Latem, Belgium) was used for analysis of Spoligotyping and MIRU-VNTR patterns. Dendrogram was constructed for Spoligotyping and MIRU-VNTR combined. The genetic distance was built employing the UPGMA algorithm (Unweighted Pair Group Method with Arithmetic Mean).

## Supporting Information

File S1 **Sex and age of patients and drug susceptibility profile, Spoligotyping and MIRU typing of 96** ***Mycobacterium tuberculosis*** **clinical isolates from cities in southwestern Paraná, Brazilian borders with Argentina and Paraguay.**
(XLS)

## References

[1] WHO. Global Tuberculosis Report 2013: WHO Press, Geneva.

[2] Agência de Notícias do Paraná. https://www.aen.pr.gov.br/modules/noticias/article.php?storyid=69313.

[3] Braga JU, Herrero MB, Cuellalr CM (2011) Transmissão da tuberculose na tríplice fronteira entre Brasil, Paraguai e Argentina. Cad Saúde Púb 27: 1271–1280. 10.1590/s0102-311x2011000700003 21808812

[4] Foxman B, Rilley L (2001) Molecular epidemiology: focus on infection. Am J Epidemiol 153: 1135–1141. 11415945 10.1093/aje/153.12.1135

[5] Barnes PF, Cave MD (2003) Current concepts: molecular epidemiology of tuberculosis. N Engl J Med 349: 1149–1156. 13679530 10.1056/NEJMra021964

[6] Masala S, Molicotti P, Bua A, Zumbo A (2010) Molecular characterization of Sardinian *Mycobacterium tuberculosis* isolates by IS6110 restriction fragment length polymorphism, MIRU-VNTR and rep-PCR. New Microbiol 33: 155–162. 20518277

[7] Supply P, Mazars E, Lesjean S, Vincent V, Gicquel B, et al (2000) Variable human minisatellite-like regions in the *Mycobacterium tuberculosis* genome. Mol Microbiol 36: 762–771. 10844663 10.1046/j.1365-2958.2000.01905.x

[8] Supply P, Lesjean S, Savine E, Kremer K, van Soolingen D, et al (2001) Automated high-throughput genotyping for study of global epidemiology of *Mycobacterium tuberculosis* based on mycobacterial interspersed repetitive units. J Clin Microbiol 39: 3563–3571. 11574573 10.1128/JCM.39.10.3563-3571.2001 PMC88389

[9] Molhuizem HOF, Bunschoeten AE, Schouls LM, Van Embden JDA (1998) Rapid detection and simultaneous strain differentiation of *Mycobacterium tuberculosis* complex bacteria by spoligotyping. Methods Mol Biol 101: 381–394. 9921492 10.1385/0-89603-471-2:381

[10] Brudey K, Driscoll JR, Rigouts L, Prodinger WM, Gori A, et al (2006) *Mycobacterium tuberculosis* complex genetic diversity: mining the fourth international spoligotyping database (SpolDB4) for classification, population genetics and epidemiology. BMC Microbiol 6: 6–23. 16519816 10.1186/1471-2180-6-23 PMC1468417

[11] Santos ACB, Gaspareto RM, Viana BHJ, Mendes NH, Pandolfi JRC, et al (2013) *Mycobacterium tuberculosis* population structure shift in a 5-year molecular epidemiology surveillance follow-up study in a low endemic agro-industrial setting in São Paulo, Brazil. Int J Micobacteriol 2: 156–165. 10.1016/j.ijmyco.2013.06.003 26785984

[12] Noguti EN, Malaspina AC, Barreto AC, Hirata RDC, Hirata MH, et al (2010) Genotyping of *Mycobacterium tuberculosis* isolates from a low-endemic setting in northwestern state of Paraná in Southern Brazil. Mem Inst Oswaldo Cruz 105: 779–785. 20944992 10.1590/s0074-02762010000600008

[13] Mendes NH, Melo FA, Santos AC, Pandolfi JR, Almeida EA, et al (2011) Characterization of the genetic diversity of *Mycobacterium tuberculosis* in São Paulo city, Brazil. BMC Res Notes 29: 269–277. 10.1186/1756-0500-4-269 PMC3160979 21801364

[14] Sharma P, Chauhan DS, Upadhyay P, Faujdar J, Lavania M, et al (2008) Molecular typing of *Mycobacterium tuberculosis* isolates from a rural area of Kanpur by spoligotyping and mycobacterial interspersed repetitive units (MIRUs) typing. Infect Genet Evol 8: 621–626. 18567544 10.1016/j.meegid.2008.05.002

[15] Kovalev SY, Kamaev EY, Kravchenko MA, Kurepina NE, Skorniakov SN (2005) Genetic analysis of *Mycobacterium tuberculosis* strains isolated in Ural region, Russian Federation, by MIRU-VNTR genotyping. Int J Tuberc Lung Dis 9: 746–752. 16013769

[16] Kent PT, Kubica G (1985) Public Health Mycobacteriology: a guide for a level III laboratory. Center for Disease Control and Prevention, Atlanta.

[17] Brasil, Minist?rio da Sa?de (2008) Manual Nacional de Vigilância laboratorial da tuberculose e outras micobactérias, Brazil. 434.

[18] Cardoso RF, Cooksey RC, Morlock GP, Barco P, Cecon L, et al (2004) Screening and characterization of mutations in isoniazid resistant *Mycobacterium tuberculosis* isolates obtained in Brazil. Antimicrob Agents Chemother 48: 3373–3381. 15328099 10.1128/AAC.48.9.3373-3381.2004 PMC514764

[19] Dale JW, Brittain D, Cataldi AA, Cousins D, Crawford JT, et al (2001) Spacer oigonucleotide typing of *Mycobacterium tuberculosis*: recommendations for standardized nomenclature. Int J Tuberc Lung Dis 5: 216–219. 11326819

[20] Supply P, Allix C, Lesjean S, Cardoso-Oelemann M, Rüsch-Gerdes S, et al (2006) Proposal for standardization of optimized mycobacterial interspersed repetitive unit-variable-number tandem repeat typing of *Mycobacterium tuberculosis* . J Clin Microbiol 44: 4498–4510. 17005759 10.1128/JCM.01392-06 PMC1698431

[21] Mazars E, Lesjean S, Banuls AL, Gilbert M, Vincent V, et al (2001) High-resolution minisatellite-based typing as a portable approach to global analysis of *Mycobacterium tuberculosis* molecular epidemiology. Proc Natl Acad Sci USA 98: 1901–1906. 11172048 10.1073/pnas.98.4.1901 PMC29354

[22] Hunter PR, Gaston MA (1988) Numerical index of the discriminatory ability to typing systems: an application of Simpson's index of diversity. J Clin Microbiol 26: 2465–2466. 3069867 10.1128/jcm.26.11.2465-2466.1988 PMC266921

[23] Demay C, Liens B, Burguière T, Hill V, Couvin D, et al (2012) SITVITWEB-a publicly available international multimarker database for studying *Mycobacterium tuberculosis* genetic diversity and molecular epidemiology. Infect Genet Evol 12: 755–766. 22365971 10.1016/j.meegid.2012.02.004

